# Supplementary material for: Are carbon emissions trading and green financial instruments synergistic? -Comprehensive quantitative research based on content analysis
Source: PLoS One. 2024 Mar 7;19(3):e0298601. doi: 10.1371/journal.pone.0298601 (PMC10919601; doi:10.1371/journal.pone.0298601)
Supplement: S1 Data — (ZIP) [file pone.0298601.s001.zip › Data/地级市Z_F工作报告.zip!/║■─╧/│╗╓▌╩╨/└┤╫╘╣1⁄2╓┌║┼í╛┬φ┐╦╩2╛▌═°í┐_2015.docx]

各位代表：

现在，我代表市人民政府向大会报告工作，请予审议，并请市政协各位委员及其他列席人员提出意见。

一、2014年工作回顾

过去一年，全市上下在市委的坚强领导下，牢牢把握“稳中求进、改革创新、又好又快”的总基调，按照“一化两城三创四大”总体思路，积极应对经济下行压力增大等困难和挑战，着力稳增长、调结构、促改革、惠民生，经济社会总体呈现持续健康发展态势。

——经济增长稳中有进。预计(下同)，地区生产总值1863亿元，同比增长10.5%；公共财政收入240.4亿元、地方财政收入171.7亿元，分别增长11.2%、17.2%。投资结构趋优、增速较快。固定资产投资1820亿元，增长23.5%，三次产业投资比调整为6.8:43.4:49.8。941个重大项目完成投资1267.5亿元。107国道提质改造、268公里“十二五”干线公路续建、卜里坪路、南岭大道南段等173个基础设施项目，远大住工、格兰博一期、丰越环保等275个产业项目，曹家坪棚户区改造一期等226个民生和社会事业项目，重金属污染治理等65个节能环保项目竣工或超额完成年度投资计划。郴州机场、莽山水库等重大项目前期工作取得突破性进展。各类融资239.5亿元，报批建设用地956.2公顷。消费需求趋旺、贡献增大。社会消费品零售总额707亿元，增长12.9%，消费拉动经济增长近5个百分点。“市场建设三年行动计划”任务基本完成，市城区14个城市综合体、4个大型综合市场、6个专业市场建设进展顺利，农贸市场标准化改造98个。建成平价商店97家、农产品直销店90家。成功举办2014年中国郴州国际(温泉)休闲旅游文化节、省第五届旅游产业博览会、第二届湘南承接产业转移投资贸易洽谈会暨首届湘南工业装备博览会等大型节会，湖南会展次中心地位已经形成。

——发展活力明显增强。坚持以改革促开放、以承接促转型，取得了明显成效。重点改革深入推进。认真贯彻落实中央、省委和市委全面深化改革各项部署，实施9大类48项改革和15项改革试点，重点推进7大类20项改革。商事登记制度改革、“三证合一”登记管理试点全面推进，新登记企业数、注册资本分别增长101.7%、130.5%。非公有制经济加快发展。深化行政审批制度改革，市本级合并、取消、下放行政许可项目92项，减幅57.4%；审批服务时限压缩65.6%。财税体制、投融资体制等改革深入推进，统筹城乡“六项改革”加快实施。食品药品监管体制改革基本完成。市、县政府机构改革全面推进。开放承接再创佳绩。郴州铁路口岸正式运行，中远泓广物流园公共保税仓获批建设，广州港郴州内陆港挂牌运行，郴州至广东黄浦“五定”班列试运行，我市纳入广东往来港澳公路跨境快速通关改革试点城市。新建标准厂房283万平方米，出租率95%；承接产业转移项目300多个，宝石产业园、新能源汽车、雅居乐等一批带动性强的重大项目落户。设立湘粤开放合作试验区工作积极推进。实际利用外资11.5亿美元、到位内资343亿元，分别增长15%、10%；与央企对接新增到位资金58亿元。外贸进出口总额42亿美元、加工贸易额32亿美元，分别增长10.5%、19.3%。

——产业结构更趋优化。三次产业结构比调整为9.5：57.1：33.4，万元GDP能耗下降5%。农业现代化水平提升。耕地保护进一步加强，农业标准化生产面积261.2万亩，粮食总产量187万吨，收购烟叶100万担。新增现代农业示范园区11个、省级农业龙头企业12家、农民专业合作社1000家、省级五星级休闲农庄9家，培育年产值过10亿元农业龙头企业1家。创建国家级畜禽水产养殖示范场8个、休闲渔业示范基地2个。流转农村土地506万亩。推广农机具5.76万台(套)。新型工业化进程加快。工业技改投资增长28.1%，新增规模工业企业68家，规模工业增加值增长11.8%，总量达到1083亿元。有色金属产业“五个一”战略体系建设加快推进，湖南有色金属(郴州)指数网站上线、指数研究中心成立，国家有色贵重金属质检中心通过评审，金贵银业成功上市。新认定国家高新技术企业12家，高加工度工业、战略性新兴产业、高新技术产业增加值分别增长24.8%、18.6%和32.7%。园区规模工业增加值增长15.3%，工业集中度同比提高17.5个百分点。郴州高新区提前一年实现“千亿园区”目标。桂阳工业园获批国家级循环化改造示范试点园区。稀贵金属和LED产业列为省级创新型产业集群。住宅产业化迈出实质性步伐。新增中国驰名商标4 件、国家地理标志保护产品2件。淘汰落后产能187.3万吨，关闭“六小企业”80户、煤矿65个。现代服务业发展提速。服务业增加值增长11.2%。“中国温泉之城”建设和旅游新“五个一”工程扎实推进，新增国家4A级景区3个，接待游客人数、旅游总收入分别增长18.7%和23.9%。创建金融生态良好城市进展顺利，郴州、汝城农商行挂牌开业。金融机构各项存贷款余额分别增长10.7%和15.4%。公路水路客货运周转量、邮电业务量分别增长18.1%和37.5%。电子商务蓬勃发展，支付宝支付额居全省第二位。房地产开发投资、商品房销售面积分别增长9.8%和18.3%。

——城乡一体化进程加快。城乡规划体系不断完善。专业(专项)规划编制扎实推进。市城区控规覆盖率98%。“大十字”城镇群总体规划实施，城镇体系、土地利用和各县城总规修改及村镇规划编制进程加快。城镇发展提速提质。市城区实施基础设施项目227个，完成投资198.8亿元。“两供两治”工程加快推进，东江引水工程开工建设，城镇生活垃圾无害化处理率99.1%、污水处理率89.4%。改造城中村17个、背街小巷30条，拆除违法建(构)筑物41.7万平方米。主要街道建筑立面改造基本完成。新增新能源公交车310台，万华等4个一(二)级客运站、锁石桥公交枢纽站和公共自行车租赁(一期)工程完成。市城区绿化覆盖率、绿地率分别达42.4%和40.2%，人均公园绿地面积11.06平方米。“创卫”通过国家专家组技术评估，“创文”获得提名资格。“大十字”城镇群794个投资过亿元的“五同”重大建设项目完成投资1071亿元。资兴获批全国新型城镇化综合试点市。新增3个省级园林县城。17个镇列入全国重点镇。全市城镇化率48.5%。新农村建设有力推进。完成农村公路建设541公里、大中修134公里。农村客运公交化改造全面启动，97.3%的行政村通客运班车。各类水利投资52.6亿元，治理病险水库94座，解决农村38.9万人的饮水不安全问题。桂东沤菜水库竣工蓄水。农网改造升级扎实推进。强力规范农村村民建房，持续开展农村环境综合整治，安仁等5县市实施整县市推进，全市40%的行政村实现“三有三无”。建成省级美丽村庄8个。扶贫攻坚步伐加快。精准化扶贫措施得到有效落实，罗霄山片区扶贫项目完成投资165亿元，106国道桂汝扶贫攻坚示范带建设加快推进。6.6万人脱贫。北湖、临武列入国家富民强县试点县。11个县市区全部纳入原中央苏区县范围。

——生态质量持续提升。持续推进城乡绿化攻坚，投入造林绿化资金44.5亿元，荒山荒地造林及迹地更新55万亩，公路通道绿化1506公里，森林覆盖率提高到66%。成功创建国家森林城市。新增2个国家森林公园。全国水生态文明城市建设试点工作方案获批，东江湖水环境保护、三十六湾及陶家河流域、翠江流域综合治理强力推进。完成省政府“一号重点工程”项目65个、重金属污染治理项目44个，跨境出市断面水质明显提高。西河生态休闲风光带建设顺利推进。郴资桂“两型社会”示范带重大项目完成投资744.5亿元，永兴、宜章纳入省两型社会建设示范点。桂东、汝城、宜章、临武、嘉禾列为国家主体生态功能区国家级示范试点。创建国家级生态乡镇3个、省级生态乡镇18个。我市被列为全国生态文明示范工程试点市，并荣获“首批创建生态文明典范城市”称号。

——社会发展和谐稳定。“民生100工程”年度任务全面完成，财政民生支出占总支出73.6%。城镇和农村居民人均可支配收入分别为23580元、10816元，分别增长9%和11.6%。新增城镇就业6.8万人、农村劳动力转移就业4.8万人，城镇失业登记率3.96%。开工建设保障性住房和棚户区改造3.86万套，改造农村危房1.3万余户。新改建乡镇敬老院10所。城镇居民医保人均补助、城乡低保和基础养老金、农村五保供养对象等补助标准进一步提高。市城区和县城义务教育学位紧张状况进一步缓解，“学前教育三年行动计划”基本完成，职业教育加快发展。郴州师范升格为湘南幼儿师范高等专科学校。全市专利授权771件，苏仙、宜章列为省知识产权重点县。市级区域卫生信息化平台建设加快推进，疾病控制和卫生应急水平明显提升，被授予“全国无偿献血先进城市”称号。食品药品安全得到有效保障。解决广播电视盲村用户收视问题4.4万余户。积极推进历史文化名城保护，新增3个国家非物质文化遗产项目。大力开展生育关怀行动，全面落实“单独两孩”政策和“城独”奖励政策，人口自然增长率6.64‰。积极创建体育之城，推动全民健身，成功举办国际女子排球精英赛、中国之队国际足球赛等多项国际性体育赛事。第三次经济普查工作获评全国先进。新闻出版、民族宗教、外事侨务、统计、贸促、海关、检验检疫、档案、地震、水文、气象、科普、地方志、老龄、慈善和工会、妇女、儿童、残疾人等事业取得新进步。落实优抚安置政策，支持驻郴部队建设，国防动员和后备力量建设扎实推进，新型军政军民关系更加巩固，驻郴部队为地方发展稳定作出新贡献。安全生产形势总体平稳，被评为全省安全生产工作先进单位。公路“限货治超”模式全省推广。法治郴州、平安郴州建设深入推进。加强和创新社会治理，深化“打黑除恶”等专项斗争，建成4个全国和谐社区建设示范社区，侯卫东官场笔记 综治民调连续5年居全省榜首，综治工作被评为全省先进市。

在推动经济社会发展的同时，市政府切实加强自身建设，全面推进依法行政，确保各级行政管理机关依照法定权限和程序行使职权、履行职责。扎实开展党的群众路线教育实践活动，深入推进作风大整顿，切实加强廉政建设，严格行政效能监察，查处了一批违纪违法案件，集中解决了一批事关群众切身利益的突出问题。深化政务公开，有序推进电子政务建设，严格落实市政府负责人接听市长公开电话、接待信访群众并督办落实机制，全面实施综合绩效考核，政府执行力持续提升。加强审计监督、财政投资评审和预算绩效管理，全面推行部门预决算和“三公”经费预决算公开。认真执行市人大及其常委会决议决定，自觉接受市人大及其常委会的法律监督、工作监督和市政协的民主监督，扎实办理人大代表建议和政协提案。注重发挥各民主党派、工商联、无党派人士和人民团体的参政议政作用，科学民主决策水平持续提高。

各位代表!过去的一年，我们面临经济下行的困难超乎预料，付出的努力非同寻常，取得的成绩有目共睹，实现了“大干新三年”的圆满收官。通过“大干新三年”，“三创四大”活动取得了重大成果，城市形象和综合竞争力得到了大幅提升，在很多方面创造了“郴州经验”、产生了“郴州效应”，得到了群众的普遍认同、省委省政府的充分肯定和媒体的广泛推介。今天的郴州，正以崭新的姿态向国际化现代化开放城市迈进!

这些成绩的取得，离不开省委、省政府和市委的正确领导，得益于各级人大、政协的监督支持，凝聚了全市人民的智慧和汗水。在此，我代表市人民政府，向全市各族人民，向人大代表、政协委员，向各民主党派、工商联、无党派人士和人民团体，向驻郴部队官兵，向中省驻郴单位，向离退休老同志，向所有关心支持郴州发展的各界朋友，表示衷心的感谢并致以崇高的敬意!

回顾一年来的工作，我们深切地体会到：要把握新常态下的新机遇，应对新常态下的新挑战，实现新常态下的新发展，就必须坚持科学发展不动摇，加快转变方式、调整结构，持之以恒抓转型；必须坚持解放思想不停步，继续先行先试、敢闯敢试，攻坚克难抓改革；必须坚持扩大内需不松劲，勇于务实担当、主动作为，凝聚合力抓项目；必须坚持开放合作不懈怠，始终敞开大门、主动对接，注重实效抓承接；必须坚持执政为民不偏向，主动简政放权、惠民便民，全心全意抓民生。

我们也清醒地看到，政府工作中还存在一些困难和问题，主要是：支撑发展的重大项目特别是产业项目不多，土地、资金、人才等要素制约仍然突出，部分企业生产经营困难，创新驱动能力不强，经济下行压力持续加大；财政持续增收基础不牢固，财税收入结构有待优化，财政收支矛盾突出；城乡居民收入增长放缓，安全生产、环境保护、食品药品安全、教育、卫生等事关群众切身利益的问题较多，保障和改善民生与群众的期盼差距较大；一些地方和单位的干部出现了松劲、懈怠情绪，“为官不为”现象仍然存在。对此，我们将采取有效措施，切实加以解决。二、2015年工作安排

今年，是郴州撤地建市20周年，也是“十二五”规划的收官之年。政府工作的总体要求是：全面贯彻党的十八大和十八届三中、四中全会精神，牢牢把握稳中求进的工作总基调，主动适应经济发展新常态，以全面建成小康社会为总揽，以提高质量和效益为核心，更加注重改革创新，更加注重转型发展，更加注重生态建设，更加注重惠及民生，更加注重依法行政，推动经济平稳健康发展和社会和谐稳定。主要预期目标是：地区生产总值增长10%以上；规模工业增加值增长11.5%；固定资产投资增长22%；社会消费品零售总额增长13%；公共财政收入和地方财政收入增长10%以上；城镇居民人均可支配收入、农民人平纯收入分别增长8%和10%以上；城镇登记失业率控制在4.6%以内；人口自然增长率控制在8.1‰以内；单位GDP能耗下降5.96%，完成省里下达的减排目标。在此基础上，要集中力量开展“新三创”活动、推进“五大提质”、抓好“五大工程”、举办“五大节会”、实现“五大突破”。围绕上述要求和目标，主要抓好八个方面的工作。

(一)全面深化改革，强化创新驱动。坚持问题导向，突出改革重点，扎实开展“改革突破年”活动，以重点突破带动全局改革。推进经济体制改革。创新重点领域投融资机制，加快推出一批非国有资本投资项目。深入推进金融改革创新，统筹推进各县市农信社改革，抓好农村产权抵押融资改革等试点。建立部门预算专项资金项目绩效目标管理机制，探索创新财政资金竞争性分配机制。认真抓好预算管理制度、税收制度及非基本公共服务、资源、环保等价格改革。深化农村电网管理体制改革。积极稳妥推进土地管理制度改革。加快政府融资平台转型发展，防范债务风险。推进行政管理体制改革。深入推进简政放权和行政审批制度改革，动态调整和精简审批事项，推进行政审批流程标准化建设，实施重大投资项目跨部门协同办理，提高行政审批效率。全面推进商事登记制度改革和“三证合一”登记管理，更加注重事中事后监管。健全市政务服务中心运行与管理机制，完善政务服务体系。加快完善全市统一的公共资源市场交易体系。深化行政执法体制改革，积极探索综合执法。统筹推进其它改革。加快事业单位分类改革。全面推进县级公立医院综合改革。深化医保付费方式改革。深化教育领域综合改革，推进各类教育协调发展。加快户籍制度改革，全面放开建制镇和小城市落户限制。深化社会治理和司法体制改革。全面推进公务用车制度改革。推进科技创新发展。大力实施创新驱动战略，加快科技创新平台建设，完善科技创新体系。突出企业创新主体地位，大力支持中小微企业开展技术创新。实施一批重大科技专项，引导和支持稀贵金属、LED、石墨等产业龙头企业联合开展共性和关键技术攻关。探索推进科技成果评价试点工作，推进科技与金融融合发展。在郴州高新区、郴州经开区内开展科技企业孵化器建设试点示范，力争郴州经开区创建国家级创业孵化示范基地。继续实施科技青年创新创业人才计划和高层次科技创新人才工程，着力培养和聚集各类高层次人才。大力营造创新的良好环境和浓厚氛围，让创新成为郴州科学发展的一大特色、一大品牌和一面旗帜!

(二)着力扩大内需，增强内生动力。促进投资、消费良性互动，充分发挥内需对经济增长的支撑作用。强力推进项目建设。深入推进“十二五”投资和项目建设“836”计划，力争完成固定资产投资2220亿元。全市拟安排总投资1亿元以上重大建设项目1110个，年度计划投资1532亿元。重点抓好京港澳高速郴州北互通、330公里“十二五”干线公路中期调增项目、香雪中路等249个基础设施项目；中昆总部经济园、中烟郴州烟厂和湖南烟叶复烤场异地搬迁等450个产业项目；东江湖保护、三十六湾综合治理等75个节能环保项目；东江引水工程、棚户区和城中村改造、“四馆一厅”等336个民生和社会事业项目。抓好郴州机场、桂永郴赣和靖永郴赣铁路、桂新高速及宇腾有色40万吨铅冶炼、“5080”煤化工、神华国华电厂等一批重大项目前期工作。创新投融资机制，提高直接融资比重。合理安排用地计划，强化用地保障。完善重大项目工作协调和推进机制，着力解决项目建设中的突出矛盾问题。强化政府投资项目全程监管和跟踪审计。着力扩大消费需求。深化收入分配制度和事业单位工资、津补贴制度改革，完善最低工资标准调整机制，依法保障劳动者基本权益，保护合法收入，增加城乡居民特别是低收入者收入。促进房地产业持续健康发展，稳定住房消费；巩固汽车等大宗消费；扩大移动互联网等信息消费；拓展健康、家政、养老等新兴消费。实施“市场建设新三年行动计划”，加快城市综合体、综合市场、专业市场建设和农贸市场标准化改造，全面启动特色商贸城镇、商业街建设。加强农产品流通骨干网、商贸物流园区和标准化配送中心建设。举全市之力办好第三届国际矿物宝石博览会、第三届湘南承接产业转移投资贸易洽谈会、2015年中国郴州国际休闲旅游文化节，借市场之力办好一批重大体育赛事，在拉动消费、促进会展经济发展的同时，使“林中之城、休闲之都”的城市品牌更加响亮!

(三)加快转型发展，培育新增长点。按照“市场要活、创新要实、政策要宽”的要求，扎实开展“产业建设突破年”活动，精准施策、精准发力，提质升级传统产业，发展壮大战略性新兴产业，着力培育“十大新经济增长点”。推进现代农业向集约化、产业化发展。加快转变农业发展方式，落实强农惠农政策，夯实农业稳定发展的基础。扎实抓好“米袋子”“菜篮子”工程，推进农业标准化生产，确保粮食总产量持续稳定。全面完成粮食仓储设施建设和改造任务，确保粮食安全。提质现代烟草农业，收购烟叶100万担。实施“十企百社千场”现代农业发展工程和“十片百园千名”农业科技推广工程，扶持现代农业示范园区、农产品加工园和农业龙头企业、农民专业合作社、高标准家庭农场发展。继续实施“一县一品”“一乡一品”工程，支持特色优势产业形成规模。引导农村土地经营权有序流转，大力发展现代庄园经济，建设一批现代精品农庄。加强农产品质量安全监管、动物疫病防控和新型农机具推广。推进现代工业向精深化、集群化发展。持续推进有色金属产业“五个一”战略体系建设，突出发展精深加工，促进有色金属产业向价值链高端发展。重点推进永兴有色金属产业整合提升，加快桂阳工业园有色科技产业园建设。关闭限期退出的煤矿，启动煤矿自然淘汰机制，加快保留煤矿技改扩能和提质达标。大力培育电子信息、新材料、新能源、先进制造、节能环保、文化创意、生物医药等战略性新兴产业。深入推进“十城万盏”试点示范，支持半导体照明骨干企业做大做强，促进LED产业集群化发展。用好省里扶持宝石产业发展政策，加快郴州宝石产业园建设，推进宝石产业加快发展。支持远大住工加快发展，打造区域性住宅产业制造中心、研发中心。加快化解过剩产能，大力发展环保产业和循环经济，积极创建省级循环经济示范城市。推进现代服务业向高端化发展。把服务业作为全市经济转型升级的主导性产业、全面小康建设的支撑性产业来抓。加快推进“中国温泉之城”和“自驾自助游示范基地”建设，完善休闲旅游产品体系。以智慧旅游为突破口，推动旅游公共服务体系建设。加大城市旅游形象推广力度。推进苏仙岭-万华岩创建国家5A级景区、小埠创建国家4A级景区，启动莽山创建国家级旅游度假区工作。强化金融支持经济转型升级和小微企业健康发展。加强社会信用体系建设，继续创建金融生态良好城市。积极发展资本市场，引导企业兼并重组，大力推进企业上市工作。加快一批重点物流园区建设，引进和培育壮大物流龙头企业，促进现代物流业加快发展。推进房地产业持续健康发展，拓展工业地产、旅游休闲地产、养老地产。大力发展电子商务等信息服务业。大力发展体育产业，以及健康、家政、养老等新型服务业态。力争服务业增加值增长11.5%。具有郴州特色的现代服务业，将成为我市现代产业体系中最重要的支撑!

(四)扩大开放合作，提高承接水平。抢抓国家推进长江经济带建设和设立广东自贸区等战略机遇，用好省委、省政府赋予郴州的先行先试政策，坚持以产业承接促进产业转型，扎实推进湘南承接产业转移示范区建设，实质性推动湘粤开放合作试验区建设。完善提升开放平台。加快推进“两平台一基地”和口岸物流中心、铁路口岸配套、国际快件监管中心优化转型、电子口岸等建设，拓展口岸国际业务。加快往来港澳公路跨境快速通关体系建设，争取郴州铁路口岸列入国家口岸开放规划，争取国家在郴州设立进境水果指定口岸、进口肉类指定口岸。鼓励和引导权威的第三方检测实验室在郴设立省级以上区域总部或分支机构。融合各类平台优势，探索发展平台经济，实现项目集中、资源集约、功能集成、企业集聚、产业集群。提升园区承载功能。深入推进“产城融合”，开展“园区建设攻坚年”活动，实施“555”工作计划，不断提高园区经济集中度和发展层次，使园区成为投资创业的主战场、新型工业的主平台、财税增长的主源泉。加快完善园区基础设施和公共服务配套，提高园区综合承载能力。大力推进园区创新发展，吸引更多具有实力的投资者。抓好宜章经济开发区“飞地经济”试点。确保郴州高新区成功创建国家级高新区，力争郴州出口加工区转型升级为国家综合保税区。提高招商引资实效。创新招商理念和方式，突出选商引资，瞄准世界500强企业、大型央企、百强民企和上市公司，着力引进大企业、创新型企业和大项目、高新技术项目，提升招商引资层次和水平。加强招商项目跟踪服务，提高招商引资实效。力争实际利用外资、到位内资均增长15%以上。大力发展对外贸易。重点培育和扶持年进出口1000万美元以上出口导向型行业龙头企业。大力发展跨境贸易电子商务，力争获批跨境电子商务试点。开放合作、主动对接，将为我们在新常态下实现新发展，赢得更多的机遇和广阔的空间!

(五)着眼城乡统筹，促进一体化发展。坚持以新型城镇化为总揽，统筹推进城乡、区域协调发展。加强规划引领。加快规划体制改革，健全空间规划体系，完成城市总体规划修改，抓好城市住房建设规划、排水防涝综合规划、“空港新城”概念性规划、轨道交通线网规划编制和下湄桥西片区控规修改。积极开展县域和村镇规划制定工作，完成全市历史文化名村、镇(乡)域村镇布局、镇区(集镇)规划，强力推进以建制村为单元的村庄规划编制。强化规划依法管理，切实维护规划的严肃性和权威性。提升城市品质。以“创文”为总揽，开展新一轮城市大提质。实施城市建设项目212个，年度计划投资201亿元。加快推进城中村及棚户区改造，抓好郴江河下游综合治理等生态建设及环境整治项目，城区电网改造、排水设施改造(二期)、市第二和第四污水处理厂及管网配套等“两供两治”项目，武广高铁郴州西站扩容、地下综合管廊开发等公共配套项目建设。县以上城镇生活污水处理率达到90%以上、城镇生活垃圾无害化处理率达到100%。全面推进供水设施改造与建设，力争全市城市公共供水普及率达到95%、县城达到85%。新增管输天然气用户2.32万户。继续推进公共自行车租赁、直饮水工程和停车位的建设、管理与维护。加快新能源公交车更新，新增一批出租汽车。开展“绿城攻坚”活动(五期)，实施城市绿荫行动“七大工程”，打造“五大精品”。持续推进城市亮化提质。开展“数字城管提质年”活动，落实最严格的城市管理和交通秩序整治。强化城市功能配套和精细化管理，加大老旧居民小区改造力度，切实解决市民日常生活中的突出问题。大力发展城市经济，促进城市可持续发展。彰显城镇特色。坚持组团式推进、特色化建设、差异化发展，打造一批各具特色的卫星城镇。加快推进“大十字”城镇群“五同工程”，重点抓好省级两型示范创建项目、清洁低碳试点项目建设。推进资兴国家新型城镇化综合试点建设。大力推进县城和小城镇建设，继续抓好示范镇发展，引导城镇特色错位发展。支持各县市争创国家级园林县城、卫生县城。建设美丽乡村。全力推进统筹城乡发展“十大示范工程”。继续推进农村公路养护大提质，完成200公里以上农村公路建设，实现100%建制村通水泥(油)路。加快农村客运公交化和渡船标准化改造，推进城乡客运一体化。抓好莽山水库等一批水利工程建设和305座病险水库(水闸)除险加固。解决38.56万农村人口的饮水不安全问题。推进“四美三宜二园”美丽乡村建设，持续加强农村村民建房管理，统筹农村危房改造和“空心村”整治，突出抓好小康新村建设。全力推进农网改造升级工程建设。加强少数民族特色村镇建设。大力发展县域经济和村级集体经济。大力推进扶贫攻坚。坚持因地制宜、突出特色、分类施策，大力推进以4个罗霄山片区县、498个省级贫困村为重点的精准化扶贫，实现7万人脱贫。全面推进移民避险解困搬迁工作，抓好永兴、汝城移民避险解困搬迁全国试点。以人为本、城乡一体的新型城镇化，将让全市人民的生活更加幸福美好!(六)突出综合施策，建设生态文明。坚持源头严防、过程严管、后果严惩，持续推进生态保护。全面推进生态创建活动。深入实施“青山、碧水、净土、蓝天”工程，巩固“创园”“创森”成果。大力创建国家生态园林城市、国家环境保护模范城市和全国生态文明示范工程试点市，市里重点建设郴资桂、郴永宜高品质生态景观带、“两高”沿线森林景观提质等9个生态文明示范工程，着力创建一批国家级生态县、生态乡镇、生态村。从严落实“五禁”措施，开展裸露山地绿化攻坚战，加强荒山荒地及迹地更新造林、抚育管护和重点区域绿化，全面实施禁伐区封山育林，持续推进生态廊道提质和公路通道绿化。积极推进桂东、汝城、宜章、临武、嘉禾南岭山地森林及生物多样性生态功能区国家级示范试点县建设。深入推进全国水生态文明城市建设试点，加强东江湖生态环境保护和小流域综合治理，全面完成西河生态休闲风光带建设。落实最严格的水环境保护措施，确保集中式饮用水源地水质达标率保持100%。扎实推进环境综合治理。强力推进三十六湾及陶家河流域、翠江流域环境综合治理。大力推进大气污染防治，加快淘汰机动车黄标车。加强固体废物等污染监管和防治，大力推行垃圾分类管理。继续推进农村环境综合整治，按照“户分类、村收集、乡清运、县处理”模式，全面启动农村生活垃圾三年专项治理，力争80%的行政村实现“三有三无”目标。加强畜禽养殖业污染综合治理。严格环境监管和环境执法，加强污染隐患排查整改，防止发生环境污染事件。切实抓好节能减排。加快推行合同能源管理，抓好重点企业、重点领域节能减排，实施一批节能减排工程，推进主要污染物减排工作，全面完成“十二五”主要污染物总量减排、重金属污染治理及省政府“一号重点工程”第一个“三年行动计划”的各项任务。加强资源能源节约集约利用，推动经济发展方式向绿色、集约、低碳转变，为子孙后代留下天蓝、地绿、水净的生活家园!

(七)坚持以民为本，增进民生福祉。进一步完善保障和改善民生的长效机制，促进基本公共服务均等化，不断增强人民群众的幸福感。继续实施“民生100工程”。重点在创业就业、社会保障、“两房两棚”“两供两治”、中小学校和幼儿园建设、农村公路建设、农村饮水安全、农村环境整治、农网改造、农村通信、医疗卫生、拆迁安置、社会救助、社会管理、便民服务等方面实施100个左右民生项目，预计投资150亿元以上。推进创业带动就业。开展“全民创业年”活动，激发民间投资和创业热情。加强就业创业培训，职业技能培训6.5万人。完善创业孵化基地14个。统筹做好各类群体的就业工作。举办首届创新创业人才招聘会。力争新增城镇就业6.3万人、农村劳动力转移就业3.9万人。完善社会保障体系。坚持全覆盖、保基本、多层次、可持续，统筹推进城乡社会保障体系建设。推进在校学生基本医疗保险全覆盖，完善城乡居民大病保险制度，启动实施城乡居民医疗保险省内异地住院医疗联网即时结算工作。有效落实各项养老保险政策，完善城乡居民养老保险制度。进一步探索完善失业保险稳定就业、预防失业功能。完善社会救助体系，保障特殊困难群众基本生活。加大保障性住房建设力度，规范分配和管理。全面发展社会事业。着力推进中心城区和县城义务教育扩容提质，全面实施“村小改造三年行动计划”、第二期“学前教育三年行动计划”。在推进教育均衡发展的同时，更加注重提高教育质量。加快发展现代职业教育，支持郴州职业技术学院建成省级示范性高等职业院校。加强学校及周边和校车安全管理。加快推进基层公共文化设施建设，全面完成农村广播“村村响”工程。完善公共文化基础设施和服务体系，加强历史文化名城保护，广泛开展群众文化活动，着力提升文化软实力。继续推进体育之城创建，广泛开展群众性体育运动，举办全市首届体育运动会。加强公共卫生服务体系和基层医疗卫生设施建设，开展示范乡镇卫生院创建活动，力争消除卫生室空白村。抓好重大传染病防治。扶持中医药发展。加强食品药品监管，建立基层监管网络，推进乡镇食品药品安全放心工程，确保食品药品安全。努力稳定适度低生育水平，推进生育关怀行动提质扩面，统筹解决人口问题。做好民族宗教、新闻出版、外事侨务、统计、贸促、海关、检验检疫、档案、地震、水文、气象、科普、地方志、地名普查、慈善和人民防空等工作。切实维护妇女儿童、老年人和残疾人合法权益。加强和创新社会治理。深入推进法治郴州、平安郴州建设和普法教育。加强城乡社区建设，推进社区网格化管理。完善重大项目、重大决策社会稳定风险评估机制，畅通和规范群众诉求表达渠道，全面推行社会矛盾“大调解”和“一村一社区一法律顾问”制度，妥善解决好信访热点和遗留问题。加强法律援助工作，推进社区矫正中心建设。完善立体化社会治安防控体系，严厉打击各类违法犯罪活动，确保社会治安持续稳定。加强公共安全和防灾体系建设，强化基层应急管理，增强应急救援能力。开展“安全生产提升年”活动，打好安全生产“十大战役”，确保实现“一杜绝一遏制一严控”目标。持续推进公路“治超”和客运市场整治。统筹经济建设和国防建设，积极推进军民深度融合，认真落实军人优待抚恤政策，广泛开展全民国防教育和“双拥共建”活动，进一步巩固军政军民关系，争创“全国双拥模范城”。

(八)推进依法行政，建设法治政府。全面贯彻依法治国重大方略，运用法治思维和法治方式，加快建设职能科学、权责法定、执法严明、公开公正、廉洁高效、守法诚信的法治政府，提升深化改革、推动发展、维护稳定的能力。依法全面履行政府职能。进一步理顺政府机构职责关系，着力简政放权、转变职能。坚持“法无授权不可为，法定职责必须为”，加快建立权力清单、责任清单、负面清单，营造有利于创新创业的政策环境和制度环境，创造更好的市场竞争环境。强化执行职责，坚决纠正不作为、乱作为，坚决克服懒政、怠政，坚决查处失职、渎职。进一步完善科学民主依法决策机制，提高行政决策的科学化、民主化和法制化水平。强化决策落实，严格绩效考核，确保政令畅通。坚持严格规范公正文明执法。完善和严格执行行政裁量权基准制度，细化、量化行政裁量标准，规范裁量范围、种类、幅度，规范行政许可、行政处罚、行政收费等执法行为。推行行政处罚、行政许可、行政复议决定等执法活动网上公开。加强食品药品、安全生产、环境保护、劳动保障、医疗卫生等关系群众切身利益、群众反映强烈的重点领域执法，依法严格查处各类违法行为。加强行政执法队伍建设。强化行政权力制约和监督。认真执行市人大及其常委会决议决定，自觉接受市人大及其常委会、市政协的监督，不断提高人大代表建议和政协提案办理质量，主动听取民主党派、工商联、无党派人士和人民团体的意见。完善政府内部层级监督，加强对权力集中部门和岗位的监督。深化政务公开，推进行政审批服务事项网上办理。加强行政监察，推进审计监督在公共资金预算、国有资产资源管理和领导干部履行经济责任等方面全覆盖。坚持厉行节约、反对浪费，严格控制“三公”经费和一般性支出；除涉密单位外，所有预算单位均公开部门预决算及“三公”经费预决算。巩固扩大党的群众路线教育实践活动成果，深入推进作风大整顿，进一步提升行政效能和服务质量。坚持用制度管权管事管人，以“零容忍”态度惩治腐败!

科学编制“十三五”规划是今年的一项重大任务。要从郴州实际出发，深入研究新常态下经济发展规律，准确把握机遇，积极应对挑战，科学提出今后五年的发展目标和任务，使规划成为凝聚力量、指引发展的任务书、路线图。

各位代表!新常态孕育新机遇，新目标引领新征程。让我们紧密团结在以习近平同志为总书记的党中央周围，在中共郴州市委的坚强领导下，团结和依靠全市人民，把握新常态，立足新起点，创造新业绩，为郴州全面建成小康社会而努力奋斗!
